# The Materials Provenance Store

**DOI:** 10.1038/s41597-023-02107-0

**Published:** 2023-04-06

**Authors:** Michael J. Statt, Brian A. Rohr, Dan Guevarra, Santosh K. Suram, Thomas E. Morrell, John M. Gregoire

**Affiliations:** 1 Modelyst LLC, Palo Alto, CA 94306 USA; 2 Division of Engineering and Applied Science, California Institute of Technology, Pasadena, CA 91125 USA; 3 Liquid Sunlight Alliance, California Institute of Technology, Pasadena, CA 91125 USA; 4 Toyota Research Institute, Los Altos, CA 94022 USA; 5 Caltech Library, California Institute of Technology, Pasadena, CA 91125 USA

**Keywords:** Electrocatalysis, Combinatorial libraries

## Abstract

We present a database resulting from high throughput experimentation, primarily on metal oxide solid state materials. The central relational database, the Materials Provenance Store (MPS), manages the metadata and experimental provenance from acquisition of raw materials, through synthesis, to a broad range of materials characterization techniques. Given the primary research goal of materials discovery of solar fuels materials, many of the characterization experiments involve electrochemistry, along with optical, structural, and compositional characterizations. The MPS is populated with all information required for executing common data queries, which typically do not involve direct query of raw data. The result is a database file that can be distributed to users so that they can independently execute queries and subsequently download the data of interest. We propose this strategy as an approach to manage the highly heterogeneous and distributed data that arises from materials science experiments, as demonstrated by the management of over 30 million experiments run on over 12 million samples in the present MPS release.

## Background & Summary

Two primary modalities for public release of large quantities of experimental materials science data are exemplified by (i) the Materials Data Facility^1,2^, which seeks to aggregate data from practically any materials science experiment, and (ii) the High Throughput Experimental Materials (HTEM)^3,4^ and Materials Experiment and Analysis Database (MEAD)^5^ databases, which focus on data management from high throughput experiments within a single institution. This latter type of data management has to-date been accessible through a web interface, which does not provide the requisite flexibility for a breadth of use cases. For example, specific subsets of the MEAD database have been curated^6^ to enable adoption of machine learning methods^7^, which contribute to the larger vision of transforming experimental science with modern data science tools^8–10^. Assembling such a dataset via a web interface is impractical, motivating our effort to enable a representation of the data that supports a breadth of use cases. Based on the recently reported event sourced architecture for materials provenance management (ESAMP)^11^, we have transformed the MEAD dataset, including additional data acquired since the original dataset publication, into a new database. The resulting database is the Materials Provenance Store (MPS), whose schema, contents, and usage is introduced herein. The MPS name reflects not only that it is literally a data store, but also that users may shop for a desired experimental materials provenance via PostgreSQL queries. The materials provenance refers to the entire experimental history of each material, which entails the sequence of experimental processes that are each described by metadata. The data origination as described by MEAD combined with the DBGen ingestion workflow provide the data provenance of each piece of experimental data, and the encoded sequence of experimental processes additional provides the experimental materials provenance that collectively resulted in the given piece of experimental data.

**Fig. 1:**
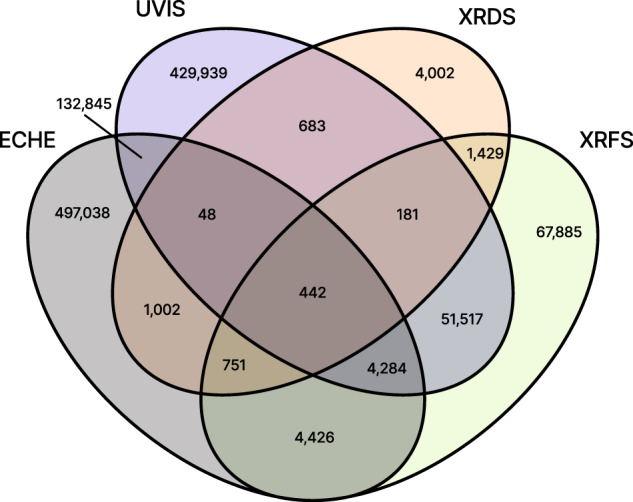
Four-way Venn diagram for the 4 primary types of experimental processes showing how many unique materials samples in the dataset have undergone each combination of process types. The process types are electrochemical characterization (ECHE), ultraviolet-visible optical spectroscopy (UVIS), x-ray diffraction (XRDS), and x-ray fluorescence (XRFS).

By modelling each experimental “Process” and its application to a given materials “Sample”, the high throughput experiments are tracked via a central “Sample Process” table, which contains ca. 30 million entries from ca. 24 million combinations of sample and process-type, a high level description of the type of experimental process. A breakdown of the number of entries for the 13 process types is shown in Table 1. Due to variability in the experimental workflows, different samples may be subject to different types of processes. A summary of the number of unique materials samples for each combination of the primary 4 process types for materials characterization is shown in Fig. 1.

**Table 1:** The 13 types of experimental processes in the database are listed with the respective number of entries in the Sample Process table.

Process	Num. Sample	Num. unique
type	Processes	Samples
print	14,351,200	11,243,172
anneal	10,464,567	9,699,800
eche	2,513,044	640,836
metr	1,104,039	942,062
imag	1,001,728	855,151
uvis	753,627	619,939
ecqe	153,092	74,923
xrfs	152,736	130,915
pets	140,800	71,424
ssrl	125,27	12,527
xrds	8,641	8,538
ecms	360	76
xtrn	7	2

Since a given sample may undergo several processes of the same type, the number of unique samples represented by each set of sample processes is also shown. The brief descriptions of process types are as follows: deposition of materials onto a substrate (print), thermal annealing (anneal), electrochemistry (eche), optical imaging for quality control (metr), imaging for colorimetric characterization (imag), ultraviolet-visible optical spectroscopy (uvis), 2-electrode photoelectrochemical characterization (ecqe), x-ray fluorescence (xrfs), parallel electrochemical treatment (pets), synchrotron x-ray diffraction (ssrl), x-ray diffraction (xrds), integrated electrochemistry and mass spectroscopy (ecms), and externally-sourced experiments (xrtn).

Batches of raw and analyzed data are stored in a separate repository, enabling a relatively small PostgreSQL database file, whose downloadable compressed size is 4.5 GB and uncompressed size is 20 GB. This file is relatively portable compared to the entire dataset, which includes 1.1 TB of compressed raw and analyzed data. These data are packaged as a matter of convenience during their generation, with each package receiving a unique digital object identifier (DOI). The 26,105 DOIs hosted by CaltechData (data.caltech.edu) are provided as a supporting document. We provide examples of programmatic access to the open-source raw and analyzed data based on the results of a given query of the MPS, demonstrating our strategy for agile data exploration and efficient utilization of the open source data repository.

## Methods

The experimental methods for generating the data are described previously, with individual implementations of these methods encoded in the Process Details table within the database. For the process types shown in Table 1, the originating process for each sample is a “print”, which includes sputter deposition from our custom Kurt J. Lesker combinatorial deposition system^12^ and inkjet printing of mixed precursors using a JetLab Microfab^13^ or C2Fast^14^ printer. The “anneal” process involves heating in a box furnace with ambient air, a tube furnace with controlled atmosphere, or a rapid thermal processing instrument^15^. A “metr” process entails optical imaging of combinatorial libraries for quality control, and “imag” entails the imaging of an individual sample for colorimetric characterization^16^. The primary materials property characterization are “eche”, electrochemical characterization in a scanning droplet cell^17^; “ecqe”, photoelectrochemical characterization with facile redox couples^18^; “uvis”, ultraviolet-visible optical spectroscopy^19^; “pets”, parallel electrochemical operation of catalyst libraries^20^; and “ecms”, electrochemical measurements with on-line mass spectroscopy data for product analysis^21^. The processes intended to characterize the composition and structure of materials include “xrds”, x-ray diffraction using a Bruker DISCOVER D8; “ssrl”, synchrotron x-ray diffraction^22^; and “xrfs”, x-ray fluorescence using a EDAX Orbis Micro-XRF. The final type is “xtrn”, which describes a process that was performed by an external collaborator.

To summarize the types of experimental provenances in the database, we briefly summarize the high throughput experiment workflows. A workflow typically commences with a “print” process wherein material is deposited onto a substrate, also known as a library plate. The material is typically reactively annealed to form a metal oxide sample via an “anneal” process. A “metr” optical imaging process is performed to ensure that material is deposited in the correct location on the library plate. From here, the workflows have considerable variability due to the different types of research being conducted with these high throughput tools. An electrocatalyst screening workflow could include an “xrfs” process to measure composition, a sequence of “eche” processes to characterize activity, and an additional “xrfs” process to see if the electrochemistry changed the composition. To discover solar light absorbers, a “uvis” process characterizes the spectral absorption with “xrfs” and “xrds” processes to characterize the composition and structure.

## Data Records

**Fig. 2:**
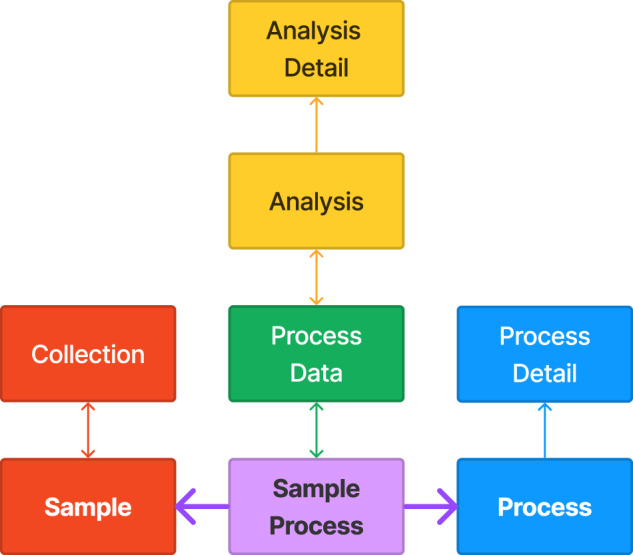
Schema diagram for the Materials Provenance Store. Each rectangle represents a database table, and each arrow represents a relationship between two tables. A single-headed arrow represents a many-to-one relationship, which is stored in the database as a foreign key. For example, the blue arrow pointing from Process to Process Detail indicates that there is a foreign key column in the Process table called process_detail_id, which references the ID column in the Process Detail table. Therefore, many rows in the Process table can be linked to one row in the Process Detail table. Each double-headed arrow represents a many-to-many relationship, which is stored in the database as a mapping table. For example, the double headed yellow arrow between Process Data and Analysis indicates that there is a mapping table (called process_data_analysis), which has only two columns: a foreign key to the Process Data table and a foreign key to the Analysis table. Tables and relationships are colored as follows: red for materials samples, blue for processes, green for process data, and yellow for analyses. The Sample Process table and its relationships, which are core to the fundamental concept of this database, are shown in purple.

**Fig. 3:**
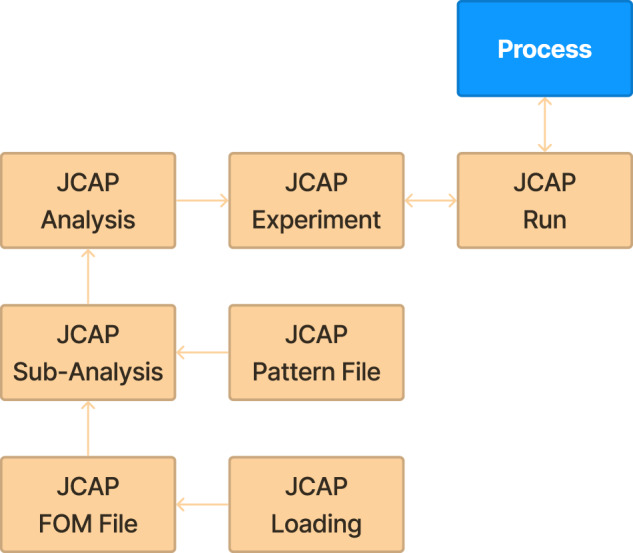
Schema diagram for the ingestion tables in the Materials Provenance Store. This figure can be read in the same way as Fig. 2. The “ingestion” tables, shown in tan, are not meant to be accessed by most users. They exist because the process of getting data into the Materials Provenance Store is quite complex, and it was useful to store intermediate intermediate linkages and results as a part of the data ingestion pipeline. Note that the JCAP Analysis table contains the DOIs for some of the underlying raw data; however, the data in these files is stored in a more accessible manner in the main tables (shown in Fig. 2).

The dataset is available from CaltechDATA^23^. Fig. 2 shows the database schema as the relationships among tables that are described below. The full schema contains additional tables that originate from the ingestion of the MEAD^5^ database, as shown in Fig. 3. This database adheres to the FAIR principles (“Findable, Accessible, Interoperable, and Reusable”). The data records are findable because a SQL query can be used to concisely and efficiently filter for records of interest. Since the database is publicly available for anyone to download, it is accessible. By using PostgreSQL, a common, free database management system that is compatible with all major operating systems, the data is interoperable. Finally, the data is reusable because the metadata for each experiment and the provenance of each sample is formally tracked, which enables users to query the database to answer a wide variety of questions as their research interests change over time.

### Sample table

A sample is an individual material whose creation is defined upon the first process in which it can be uniquely identified in the lab. When processes are applied to the sample, intentional or unintentional changes to the material may occur, but its sample number remains in tact, with its provenance being the sequence of processes applied to it.

### Processes table

A Process is any procedure that is done to a sample. This may be a step in its preparation, or it may be an experiment intended to characterize the sample.

### Sample process table

The sample-process table underlies the core concept of this database: when a sample undergoes a process, this event can generate one or many pieces of process data. There is a many-to-many relation between samples and processes because a sample can (and usually does) undergo many processes over the course of its life, and a process can be run on many samples simultaneously. This is shown in the bottom row of the diagram below; the connections between samples and processes are represented by the purple arrows, and the table names are shown in bold font.

### Process data table

When samples undergo processes, and data does result from the experiment, this output data is stored in the ProcessData table. There is row in the Process Data table for every output file from experiments done in the lab.

### Analysis table

This Process Data can be used as the input to Analyses. A row in the analysis table represents the output of a function that accepts Process Data of a certain type as an input and returns a figure of merit (abbreviated as FOM) as its output.

### Collections

Collections are simply groups of samples. Each sample in this database is printed onto a plate. Each plate contains a few thousand samples, and although samples are often analyzed independently, it is useful to keep track of which samples are on which plate.

### Process details

Processes often have some controllable parameters like the temperature or choice of solvent, etc. These input parameters are stored in the Process Detail table. Process details are stored in a separate table to make it easy to query for processes that were run with the same set of input parameters. Two of the columns in this table, named “type” and “technique,” specify the type of experiment performed. The “details” column contains a dictionary in json format that contains all of the metadata that was recorded for that experiment. This is meant to include all relevant experimental input parameters, like the solution pH or current density set point. For each type and technique, the schema of the json column is consistent across all rows. Therefore, the metadata schema for each type of experiment can be found by querying for any row in the Process Details table with the type and technique of interest.

## Technical Validation

The database entries result from high throughput experiments and analyses of the resulting data. For experimental data describing the synthesis and characterization of materials, the technical quality of the data is monitored via standard operating procedures of the instruments. A core tenet of the database presented herein is that further technical validation must be done in the context of a specific research purpose, and to avoid injection of data quality assumptions into data analysis, the database contains all raw output from the instruments to increase transparency and allow modifications to any quality control and validation algorithms. Validation of specific subsets of data are provided in previous work, typically via replication of high throughput screening results using traditional experimental methods for catalysts^14,24–26^, photocatalysts^27,28^, and integrated photoanodes^29–31^. For each of these examples, the instrument control software was written to validate metadata tracking by 2 primary methods, automated metadata recording and manual data entry with validation. Instrument settings comprise the majority of metadata, and extraction and storage of instrument settings was performed by the instrument control software, with the resulting metadata file manually checked against instrument settings after each modification to the control software. Some manual data entry was required for select instruments, most commonly entry of the sample number, whose manually entry was protected against single keystroke errors (and most multi-keystroke errors) via a checksum. The other primary type of manual data entry is numerical calibration of instrument components, most notable the reference electrode in electrochemical experiments. The lab maintains a data log of all reference electrodes and their history of calibrations to ensure continuity and validation against the entries encoded in the metadata.

## Usage Notes

The data is available in a PostgreSQL database. This format requires three steps to make use of. It also provides the ability to use SQL queries to access specific subsets of the data. This makes it easier for researchers to ask specific questions of the data. Additionally, when a researcher writes a SQL query to acess a specific subset of the data for a given project, they can simply publish the query, and which data they used is very transparent.

- Download the compressed SQL database dump file (.tar.gz format) from CaltechDATA at https://data.caltech.edu/records/4kk39-69x76^23^.
- Install PostgreSQL by following the instructions at https://www.postgresql.org/download/.
- Extract the.tar.gz file, which will yield a.sql file.
- Follow the PostgreSQL documentation to create a new database from the.sql file.

This will create a local copy of the database that we present in this work. The data can be browsed using the DBeaver user interface, and SQL queries can be written to return specific portions of the database that are of interest to the researcher.

## Supplementary information

Supporting Information

## Data Availability

The MPS database was generated using DBgen (v1.0.0a7) (https://github.com/modelyst/dbgen), an open-source framework for building scientific databases and pipelines available at https://github.com/modelyst/dbgen. A python API, a command-line interface (CLI), and a Jupyter notebook with example queries are available in the Materials Provenance Store Client repository (https://github.com/modelyst/mps-client).
